# A Photoactivatable Small‐Molecule Probe for the In Vivo Capture of Polyketide Intermediates

**DOI:** 10.1002/chem.201903661

**Published:** 2019-11-28

**Authors:** Samantha L. Kilgour, Robert Jenkins, Manuela Tosin

**Affiliations:** ^1^ Department of Chemistry University of Warwick Library Road Coventry CV4 7AL UK

**Keywords:** bioorganic chemistry, biosynthesis, intermediate capture, photoactivatable probes, polyketides

## Abstract

A photolabile carba(dethia) malonyl *N*‐acetylcysteamine derivative was devised and prepared for the trapping of biosynthetic polyketide intermediates following light activation. From the lasalocid A polyketide assembly in a mutant strain of the soil bacterium *Streptomyces lasaliensis*, a previously undetected cyclised intermediate was identified and characterised, providing a new outlook on the timing of substrate processing.

## Graphical abstract

**Biocatalysis**: A 4,5‐dimethoxy‐2‐nitrobenzyl (DMNB)‐protected probe was devised and utilised for the capture of polyketide‐derived species in bacterial fermentative cultures (see figure).

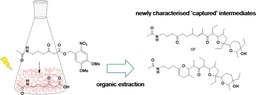

Polyketide natural products constitute an abundant family of secondary metabolites comprising prominent pharmaceuticals and agrochemicals such as fidaxomicin and avermectin.^1^ They are biosynthesised by multifunctional enzymes known as polyketide synthases (PKSs) through a common pathway: this involves multiple decarboxylative Claisen condensations of acyl carrier protein (ACP) or CoA‐bound malonyl units onto ketosynthase‐(KS) bound acyl groups (Figure 1 A). Newly generated ACP‐bound intermediates “grow” in length and chemical complexity thanks to the action of reductive enzymes (e.g. ketoreductases, dehydratases, and enoylreductases, ERs) until the end polyketide product is released from a PKS (typically by thioesterase‐, TE‐, mediated hydrolysis/cyclisation); further enzymatic tailoring ultimately afforded the mature bioactive natural product.

**Figure 1:**
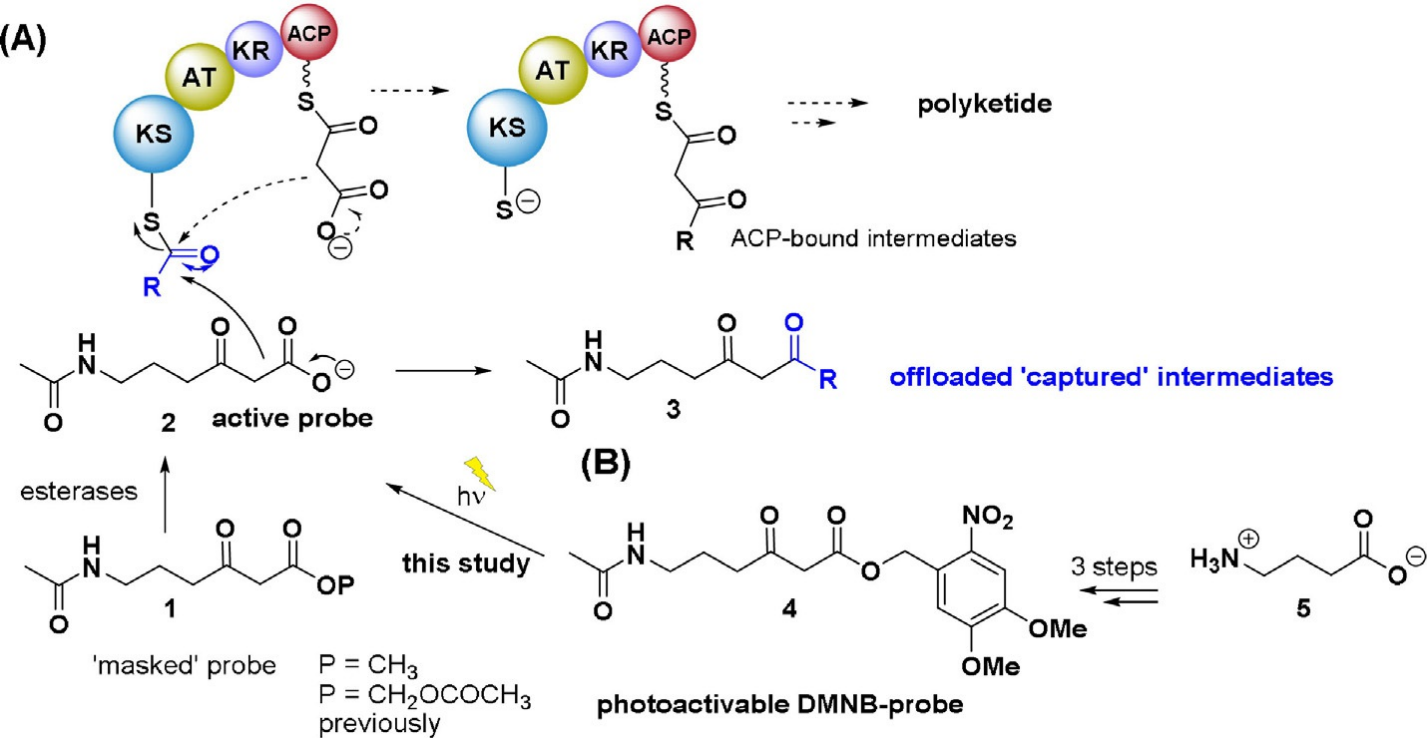
(A) Premature polyketide chain termination for intermediate capture via a nonhydrolysable carba(dethia) malonyl *N*‐acetylcysteamine **2**: this competitively interferes with polyketide chain assembly (dashed pathway) to off‐load enzyme‐bound intermediates (**3**).5a (B) Newly devised photolabile DMNB probe (**4**) for polyketide intermediate capture following light activation (*hν*). Legend: ACP=acyl carrier protein; AT=acyl transferase; KS=ketosynthase; KR=ketoreductase.

PKSs are classified as modular or iterative and into different types on the basis of their structural organisation and modus operandi.^2^ A detailed knowledge of PKS biosynthesis pathways constitutes the basis of rational enzyme engineering for the production of commodity and high value chemicals.^3^ Over time key insights into PKS workings have been provided by molecular biology, enzymology, analytical chemistry, and structural studies.3b, 4 Nonetheless challenges in PKS studies remain; these are mainly associated with limitations in our ability to closely monitor the biocatalysis in its natural context and thereby determine the exact mechanism and order of enzymatic transformations.

In our group we have devised a chemical approach for polyketide biosynthetic investigations based on the use of “chain termination” probes capable of intercepting PKS biosynthetic intermediates throughout product assembly. The probes are carba(dethia) *N*‐acetylcysteamine (NAc) derivatives that mimic the ACP‐malonyl extender units utilised in polyketide formation (**2**, Figure 1) and have been utilised for in vitro and in vivo studies to shed light on previously unknown details of complex polyketide formation, such as that of polyethers, macrolides and thiotetronates.^5^, ^6^, ^7^ More recently, the chemical chain termination strategy was successfully extended to the study of nonribosomal peptide synthetases by the use of nonhydrolysable mimics of PCP‐bound amino acids.^8^

In utilising our chemical tools for the investigation of natural product pathways in Gram‐positive bacteria, Gram‐negative bacteria and fungi, ^[7a]^ we found that chemical modifications in the probe structure greatly affect its bioavailability and intermediate capture ability in vivo. For instance, variation of the *N*‐acyl moiety of ester‐masked probes (**1**) affects both cell permeation and the extent of in vivo ester hydrolysis of **1** to generate the active probe **2**.6c Besides, the use of a labile acetoxymethyl ester as a β‐ketoacid protecting group for **2** leads to increased titres of captured intermediates, likely due to increased amounts of **2** generated in situ.6c Nonetheless tool development is still needed to improve our ability to dissect challenging biosynthetic pathways.

Amongst the most attractive approaches employed to study, control and modulate biological process is the use of light: indeed photosensitive groups do not require the use of (bio)chemical reagents for their cleavage/activation, and this last can be selectively achieved at biologically nondamaging wavelengths with high spatio‐temporal precision.^9^ Several photoactivatable moieties have been devised and employed in chemical biology for a wide range of applications,^10^ such as aryl azides, benzophenones and diaziridines to generate electrophiles for photoaffinity receptor tagging;^11^ phenacyl, benzoin and nitrobenzyl protecting groups for the ‘uncaging“ of effector/signalling molecules;^12^ and azobenzenes as conformational switches of gene expression^13^ amongst many others. In the context of natural products, photolabile ‘unnatural” amino acids have been incorporated into proteins and peptides through feeding experiments and genetic encoding for protein/peptide labelling.^14^ Furthermore, several medically relevant secondary metabolites bearing photoactive moieties have been chemically synthesised in order to identify their natural product target and mechanism of action.^15^

To the best of our knowledge, photoactivatable small molecules have yet to be utilised in the study of secondary metabolite assembly. Therefore, we decided to utilise a photolabile protecting group to mask the active β‐ketoacid moiety of our polyketide chain termination probes to establish whether 1) the group could be photolysed in vivo during live bacterial fermentation, and 2) it could be advantageous in the capture of biosynthetic intermediates. The photoactive group chosen for our studies was the 4,5‐dimethoxy‐2‐nitrobenzyl (DMNB) group, providing straightforward synthetic incorporation and cleavage upon irradiation at 365 nm, generating a low toxicity by‐product (*o*‐nitrosobenzaldehyde).^16^ The DMNB moiety has been used to protect alcohols, thiols, selenols, amines, phosphates and carboxylic acids for biological applications.^12^, ^15^, ^16^, ^17^ Various structural modifications of DMNB have been devised to increase its photolysis quantum yield; this has been shown to be dependent on the nature of the leaving group in the DMNB substrate, with esters displaying the lowest quantum yield in comparison to ethers and amines.9a

The preparation of the DMBN probe **4** (Figure 1) as the first photoactivable tool for polyketide biosynthetic investigations was accomplished from γ‐aminobutyric acid **5** in three steps (Scheme S1 and related Supporting Information). Photolysis studies of **4** in different organic solvents and buffers were performed by employing different light sources. Compound **4** could be quantitatively photolysed over a period of 2–4 hours in water by irradiation with either a KiloArc™ Broadband Arc Lamp (1000 W, 2 hours) or with an in‐house built light box containing a circular 22W UVA lamp in water (4 hours), or in MYM *Streptomyces* medium (6 hours) at similar concentrations (0.5–2.0 mm, Supporting Information). We then tested its use in vivo during the growth of *Streptomyces lasaliensis* ACP12 (S970A), a model bacterial strain extensively utilised for the investigation of the antibiotic lasalocid A assembly in our laboratories. This mutant strain, for which the last ACP of the lasalocid A synthase is inactivated, has been found to harbour several enzyme‐bound biosynthetic intermediates by us extensively characterised, and to generate unnatural polyether species such as **7** in the presence of “chain termination” probes such as **2**.^6^

Both liquid and agar‐plate cultures of the *S. lasaliensis* ACP12 (S970A) strain were supplemented with **4** (without photolysis), either in a single amount on day 1 of bacterial growth or in multiple smaller portions throughout days 2 to 5 of fermentation to achieve a similar final concentration (2.5 mm). Compound **4** was found to be almost quantitatively hydrolysed over 5 days in organic extracts belonging to “day 1” supplementation experiments, whereas it remained almost intact in samples deriving from “2‐to‐5” daily supplements (Figure S3, Supporting Information). This indicated that **4** is a relatively poor substrate for in vivo esterases, unless incubated in fermentation conditions for prolonged periods of time. In all the extracts belonging to unphotolysed samples, traces of the unnatural polyketide **7** and its linear unoxidised precursor **9** (both deriving from the off‐loading of intermediates bound to the lasalocid A synthase) were detected and characterised as previously reported (Figures S4 and S5, Supporting Information),^6^ indicating that even the presence of a small amount of **2** leads to intermediate capture. The same in vivo experiments were then carried out by subjecting the bacterial solid and liquid cultures to light irradiation via the in‐house built UVA light box (as detailed in the Supporting Information). Culture irradiation was carried out for up to 2 hours daily over a period of 5 days to minimise disruption on cell growth and metabolism.

Exposure to light irradiation did not affect cell growth and deprotection of **4** followed by decarboxylation to **2** was achieved in vivo; the extent of it varied upon culture conditions and was much more evident in liquid cultures supplemented with daily doses of **4** (Figure S6, Supporting Information). Intriguingly, the most interesting results in terms of biosynthetic intermediate capture came from the photolysis of liquid cultures supplemented with **4** in day 1. Besides the previously detected **7** and **9** generated in substantially higher amounts (Figures S7 and S8, Supporting Information), organic extract analyses by UPLC‐HRMS revealed the presence of a putative oxidised and cyclised nonaketide, possibly dehydrated (**10**, Figure 2 and Figure S9, Supporting Information). This species was characterised by HR‐MS^2^ experiments, which displayed an *m*/*z* fragment of 377 characteristic of the lasalocid A polyether part of the molecule (Figure 2 B); a possibly related species weighing 2 Da less and displaying an *m*/*z* 377 fragment was also observed (Figures S10 and S11, Supporting Information). Recent profiling of lasalocid A polyketide bio‐assembly with second‐generation chain termination probes have allowed us to off‐load and capture several putative biosynthetic intermediates, including partially and fully processed linear nonaketides such as **8** (Figure 2).6c To date only putative undecaketides and dodecaketides featuring polyether moieties had been identified; these would derive from the processing of linear enzyme‐bound polyketide chains by the tailoring enzymes LasC (an epoxidase) and LasB (an epoxide hydrolase).^6^ The characterisation of a putative nonaketide polyether species **10** in the current study suggests that either the linear nonaketide **8** is oxidised and processed by LasC and LasB following the chemical off‐loading of a fully processed PKS‐bound octaketide from module **9** or that the tailoring epoxidation–epoxide hydrolysis cascade might take place on PKS‐bound intermediate(s) earlier than previously envisaged.6a Whereas recombinant LasC and LasB are capable of processing substrate mimics of different nature and complexity,^18^ the nature of the true substrates for these enzymes in vivo remains debatable. Nonetheless, the detection of **9** and other putative captured species in specific microorganism fermentation and photolysis conditions exposes how the timing and the flux of polyketide/polyether intermediate formation is much more susceptible to local and global perturbations and still holds several intriguing aspects worthy of further investigation and exploitation.

**Figure 2:**
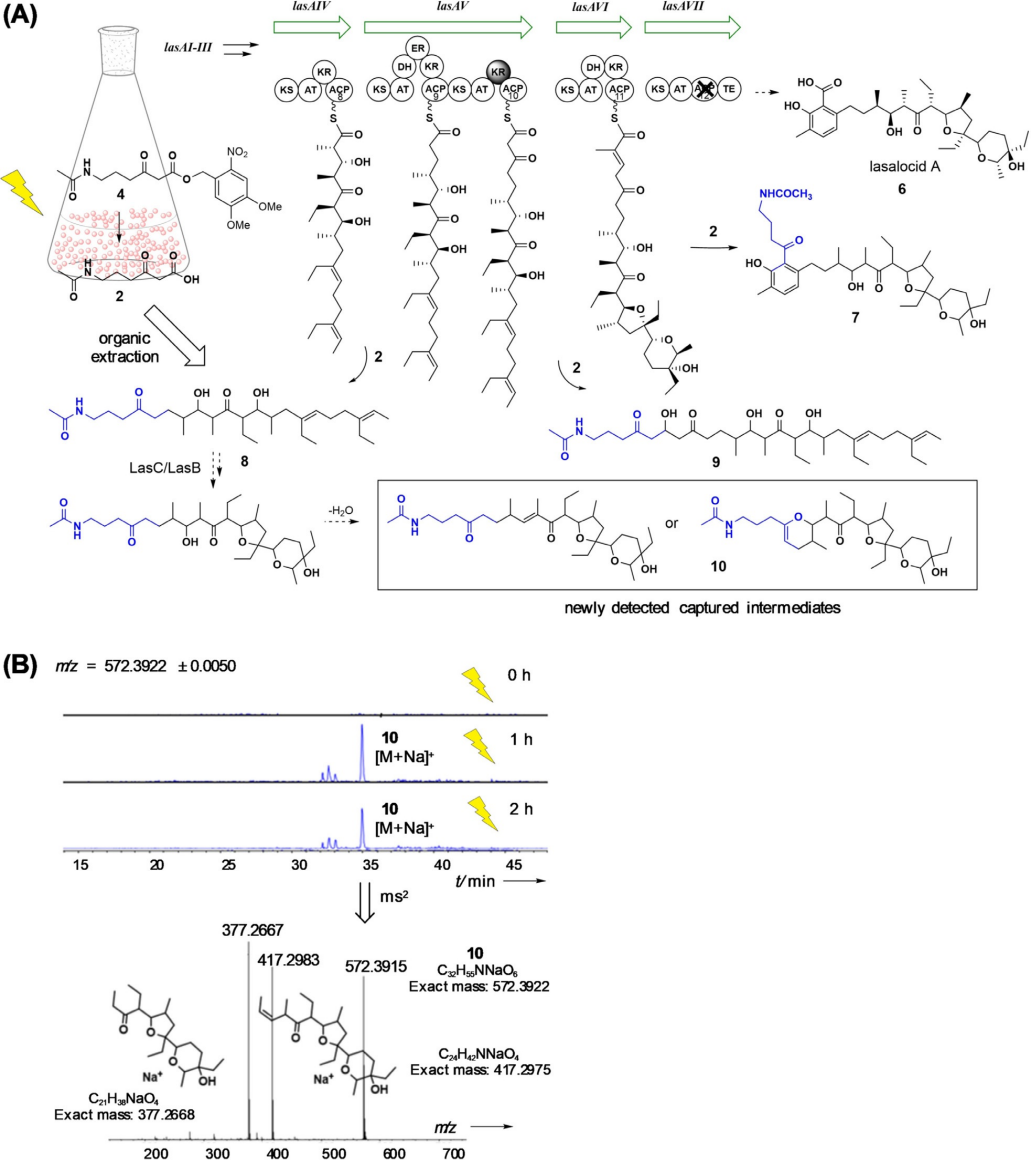
**(A)** Overview of advanced putative intermediate capture from the lasalocid A polyketide synthase during the fermentation of *S. lasaliensis* ACP12 (S970A) via the newly devised photolabile probe **4**. The stereochemistry of the putative captured species **7**–**10** has yet to be established. (B) UPLC‐HRMS^*n*^ characterisation of the newly detected putative intermediate **10** following light activation of **4**.

In summary we herein provided novel insights on lasalocid A polyketide assembly utilising a novel light‐activatable tool in vivo. Further investigations into the *modus operandi* of **4** and the development of other light‐controlled tools for biosynthetic studies^19^ holds the promise to unveil yet unknown aspects of modular as well as iterative natural product synthases.

## Experimental Section

The synthesis of **4**, its use and detailed UPLC‐HRMS^*n*^ analyses of lasalocid A captured intermediates are described in the Supporting Information.

## Conflict of interest

The authors declare no conflict of interest.

## Supporting information

As a service to our authors and readers, this journal provides supporting information supplied by the authors. Such materials are peer reviewed and may be re‐organized for online delivery, but are not copy‐edited or typeset. Technical support issues arising from supporting information (other than missing files) should be addressed to the authors.

Supplementary
