# Supplementary material for: A Photoactivatable Small‐Molecule Probe for the In Vivo Capture of Polyketide Intermediates
Source: Chemistry. 2019 Nov 28;25(72):16511–4. doi: 10.1002/chem.201903661 (PMC6972646; doi:10.1002/chem.201903661)
Supplement: Supplementary file 1 — Supplementary [file CHEM-25-16511-s001.pdf]

CHEMISTRY
A European Journal

Supporting Information

A Photoactivatable Small-Molecule Probe for the In Vivo Capture of Polyketide Intermediates

Samantha L. Kilgour, Robert Jenkins, and Manuela Tosin*[a]

chem_201903661_sm_miscellaneous_information.pdf

Contents

1 SYNTHESIS OF CHEMICAL PROBES 2

1.1 GENERAL METHODS 2

1.2 SYNTHESIS OF DMNB PROBE 4 (4,5-DIMETHOXY-2-NITROBENZYL 6-ACETAMIDO-3-OXOHEXANOATE) 3

2 PHOTOLYSIS OF 4 4

3 IN VIVO EXPERIMENTS 7

3.1 MICROBIOLOGY METHODS 7

3.2 GROWTH OF *S. LASALIENSIS* ACP12 (S970A) IN THE PRESENCE OF 4 AND MASS SPECTROMETRY ANALYSIS 7

3.3 INTERMEDIATE CAPTURE FROM *S. LASALIENSIS* ACP12(S970A) 8

4 NMR SPECTRA 14

4.1 ^{1}H- AND ^{13}C-NMR OF 4,5-DIMETHOXY-2-NITROBENZYL 6-ACETAMIDO-3-OXOHEXANOATE (4) 14

5 REFERENCES 15

1 Synthesis of chemical probes

1.1 General methods

Unless specified otherwise, chemicals were purchased from Sigma Aldrich, Fisher Scientific, Carbosynth and Alfa Aesar and were used without further purification. Anhydrous dichloromethane and toluene were purchased from VWR International (AR grade) and dried using solvent towers. Anhydrous ethyl acetate, isopropanol, butanol, dimethyl sulfoxide and pyridine were purchased from Fisher Scientific. Reagent grade dichloromethane, ethyl acetate, methanol, acetonitrile, cyclohexane, butanol and tetrahydrofuran were purchased from Fisher Scientific.

Analytical thin-layer chromatography (TLC) was performed on aluminum sheets precoated with silica gel 60 (F_{254}, Merck) and visualized under ultra-violet light (short and long-wave) using potassium permanganate ($KMnO_4$) or vanillin stains. Silica gel for flash chromatography was purchased from Sigma Aldrich (Tech Grade, pore size 60 Å, 230-400 mesh).

^{1}H and ^{13}C NMR spectra were recorded in d_4-MeOD, $CDCl_3$ or D_2O on the following Bruker Avance instruments: DPX-300 300 MHz, DPX-400 400 MHz, DRX-500 500 MHz, AV III-500 HD 500 MHz, AV-600 600 MHz or AV-700 700 MHz. UV spectra were acquired on a Varian Cary 50 Scan UV-Visible spectrophotometer.

High-resolution mass spectra (HRMS) of newly made compounds were obtained using electrospray ionization (ESI) on a MaXis UHR-TOF (Bruker Daltonics) or on Bruker MaXis (ESI-HR-MS).

Compounds were purified by semipreparative HPLC on a Phenomenex Synergi™ Polar RP 80 Å (250 x 10.0 mm, 4µm) column. The mobile phase consisted of a gradient of water (solvent A, HPLC grade) and MeOH (solvent B, HPLC grade) at a flow rate of 2.5 mL/min, with UV detection at 210, 254 and 280 nm.

1.2 Synthesis of DMNB probe 4 (4,5-dimethoxy-2-nitrobenzyl 6-acetamido-3-oxohexanoate)

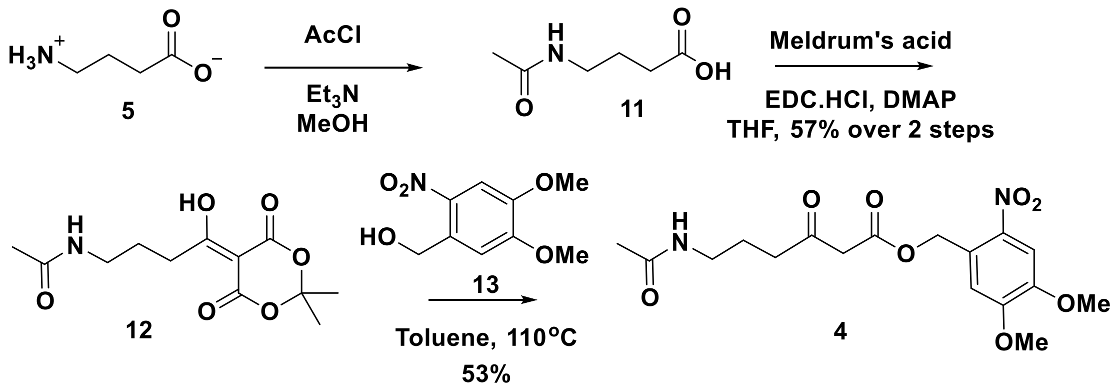

Scheme 1S: preparation of **4** from γ-aminobutyric acid (**5**)

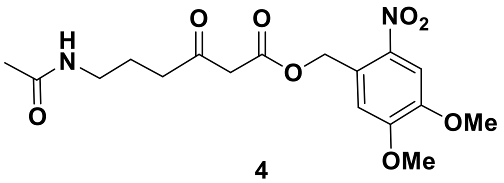

4

4,5-dimethoxy-2-nitro-phenyl)methyl 6-acetamido-3-oxo-hexanoate (4): *N*-(4-(2,2-dimethyl-4,6-dioxo-1,3-dioxan-5-ylidene)-4-hydroxybutyl)acetamide (**12**) was prepared as previously reported.[1] A solution of **12** (192 mg, 0.71 mmol) and 4,5-dimethoxy-2-nitrobenzyl alcohol (**13**, 151 mg, 0.71 mmol) in dry toluene (5 mL) was refluxed at 110°C for 16 h. The solvent was removed *in vacuo* and purification by silica column chromatography with a gradient of 0-60 % acetone in petroleum ether afforded the title compound **4** as a white solid (142 mg, 0.37 mmol, 53 %). A portion of the compound was further purified by semi-preparative HPLC (varying B as follows: from 45% to 50% in 3 minutes, to 90% over 32 minutes, back to the initial conditions in 5 minutes) for detailed NMR characterisation; R_t = 21 min. ^{1}H-NMR (500 MHz, $CDCl_3$) δ 7.73 (s, 1H, $O_2NCC\mathit{H}$), 7.14 (s, 1H, $H_3COCC\mathit{H}$), 5.62 (br s, 1H, $N\mathit{H}$), 5.60 (s, 2H, $CO_2C\mathit{H}_2$), 4.05 (s, 3H, $O_2NCCHCOCH_3$), 3.96 (s, 3H, $H_2CCCHCOC\mathit{H}_3$), 3.60 (s, 2H, $COC\mathit{H}_2CO_2$), 3.26 (q, 2H, J = 6.8 Hz, $NHC\mathit{H}_2$), 2.63 (t, 2H, J = 6.9 Hz, $NHCH_2CH_2C\mathit{H}_2$), 1.97 (s, 3H, $C\mathit{H}_3CONH$), 1.82 (quint, 2H, J = 6.8 Hz, $NHCH_2C\mathit{H}_2$); ^{13}C-NMR (100 MHz, $CDCl_3$) δ 202.6 ($CH_2\mathit{C}OCH_2$), 170.5 ($\mathit{C}ONH$), 166.7 ($\mathit{C}O_2$), 154.0 ($O_2NCCH\mathit{C}OCH_3$), 148.5

($CH_2CCHCOCH_3$), 139.8 (O_2NCC), 126.9 (O_2NC), 110.7 (CH_2CCH), 108.3 (O_2NCCH), 64.3 (O_2NCCCH_2), 56.9 ($O_2NCCHCOCH_3$), 56.6 ($CH_2CCHCOCH_3$), 49.3 ($COCH_2CO_2$), 40.6 ($NHCH_2CH_2CH_2$), 38.8 ($NHCH_2$), 23.6 ($NHCH_2CH_2$), 23.5 (CH_3CONH); HRMS: *m/z* $[M + Na]^+$, found: 405.1274, calculated: 405.1268.

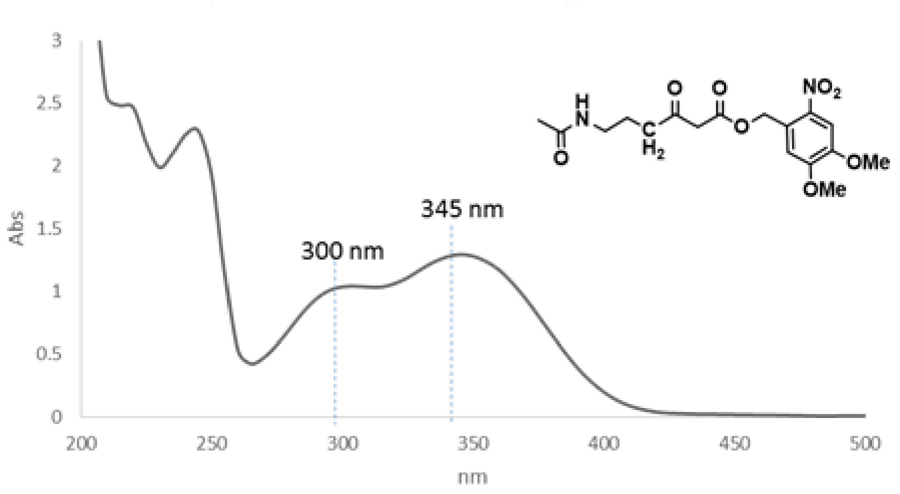

Fig. 1S UV/Vis spectrum for the photoactivatable DMNB probe **4** (c = 0.26 mg/mL in acetonitrile/water 1:1)

2 Photolysis of 4

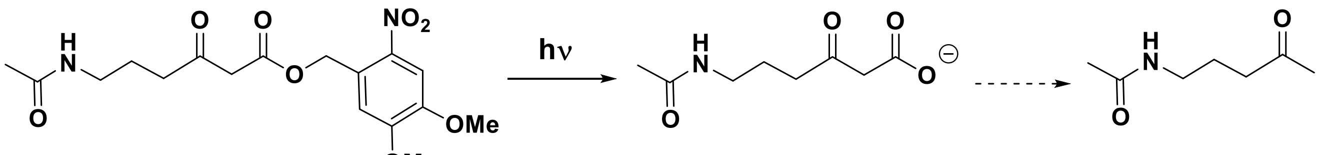

Scheme 2S: the photolysis of **4** (at 365 nm) generates the active probe **2** and ultimately carba(dethia) *N*-acetylcysteamine **14**[2]

The photolysis of **4** *in vitro* and *in vivo* was successfully carried out following the procedures detailed below. Its outcome was estimated by monitoring the formation of **2** and its decarboxylation product **14** by UPLC-HRMS of solutions/ extracts (MaXis UHR-TOF, Brucker, e.g. see Figure 2S).

In vitro: samples of **4** were placed in a quartz cuvette for irradiation with an OBB Tunable KiloArc™ Illuminator at 365 nm and 1000W. Alternatively they were irradiated within a home-built light box (purposely built by Mr Rod Wesson, Electronics workshop, Warwick Chemistry) fitted with a circular 22W UVA lamp (see details below). The samples were directly analysed by UPLC-HRMS (section 3).

Table 1S: overview of conditions for quantitative photolysis of **4**[a] *in vitro*

Light source	Sample preparation	Irradiation time
KiloArc Broadband Arc lamp 1000W, 365 nm	0.96 mM in H_2O	2 hours
In-house built light box containing a circular 22W UVA lamp	1.60 mM in H_2O	4 hours
In-house built light box containing a circular 22W UVA lamp	0.63 mM in MYM media	6 hours

[a] estimated by UPLC-HRMS analyses

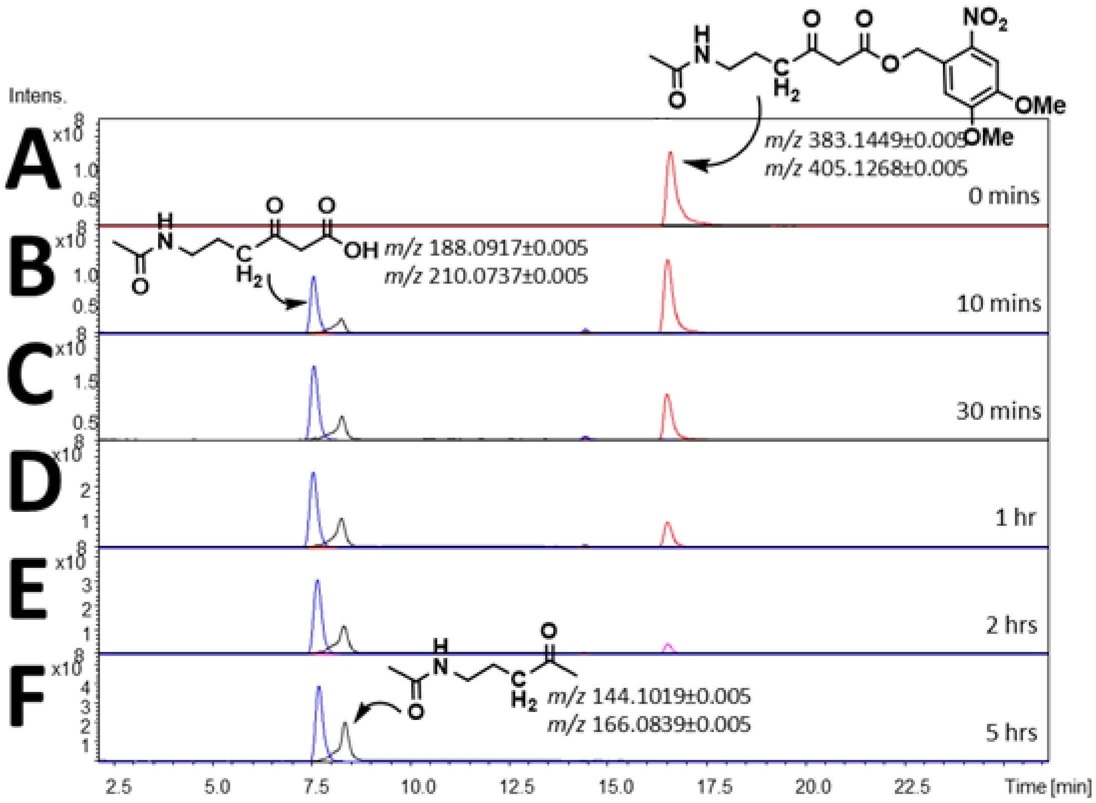

Fig. 2S Extracted ion chromatogram (UPLC-HR-MS analyses, see section 3.3) of **4** in water (~ 1 mM) and irradiated at 365 nm, 1000 W for 0 mins (A), 10 mins (B), 30 mins (C), 1 hour (D), 2 hours (E) and 5 hours (F).

<u>*In vivo*</u>: *Streptomyces* bacterial cultures in liquid or solid media containing **4** (in various amounts, up to 2.5 mM) were placed within the home-built light box, equipped with a circular 22W UVA lamp (Actinic BL TL-E 22W/10 1CT), cooling fans and an interchangeable mount for either a 5 mL petri dish or a 50 mL Erlenmeyer flask. For irradiation of shaking liquid cultures the light box was fixed to an Innova™ Incubator Shaker (New Brunswick Scientific). Cultures were irradiated for the stated time lengths (see next section).

3 *In vivo* experiments

3.1 Microbiology methods

All media and glassware were sterilized prior to use by autoclave (Astell). Liquid cultures were grown with shaking in Innova 44 incubator/shaker (New Brunswick Scientific).

S. lasaliensis ACP12(S970A) was grown and maintained as previously reported.[3] Precultures were grown in M79 medium (10 mL) at 30°C for 3 days. These (100 µL aliquots) were used to inoculate fermentation cultures in MYM (10 mL liquid or 5 mL agar plates) and grown for 5 days at 30°C.

M79 medium: 2.5 g glucose, 2.5 g peptone, 0.5 g yeast extract, 1.5 g NaCl, 2.5 g casein hydrolysate in 250 mL of tap water adjusted to pH 7.1.

MYM medium: 1.0 g maltose, 1.0 g yeast extract, 2.5 g malt extract in 250 mL of tap water adjusted to pH 7.1.

3.2 Growth of *S. lasaliensis* ACP12 (S970A) in the presence of 4 and mass spectrometry analysis

For solid cultures of *S. lasaliensis* ACP12(S970A), **4** was added to MYM agar plates during their preparation (1-2 mM final concentration). For liquid cultures, **4** was added either on the first day of fermentation in MYM, or portionwise as previously reported between days 2 and 5 of fermentation (6.25 µmol of **4**, dissolved in the minimal amount of DMSO, daily) to reach the same final concentration (2.5 mM).[4] *In vivo* photolysis of **4** was carried out as reported in section 2.

Control experiments (media alone, media without bacteria, fermentations in the absence of **4**, fermentations in the presence of **4** not subjected to photolysis) were conducted in parallel.

After 5 days of fermentation at 30°C cultures were extracted with EtOAc, evaporated and reconstituted in hplc-grade ACN/H_2O (1 mL, 1:1) for LC-MS analysis.

UPLC-HR-ESI-MS analyses of extracts were performed utilising an Eclipse Plus C18 UPLC column (1.8 µm, 2.1 x 100 mm, 0.2 mL/min flow rate) with H_2O and ACN containing 0.1% formic acid as solvents and gradients of increasing acetonitrile content (from 5% to 100% over 45-55 minutes) on a Dionex UPLC system coupled to a MaXis Impact UHR-TOF (Bruker Daltonics). Spectra were recorded in positive ionisation mode, scanning from *m/z* 50 to 2000, with Capillary Voltage set at 3500 V, Dry

Heater set at 180°C and UV Lamp set at 210 nm. Selected ion search within 5 ppm was performed, as well as high resolution fragmentation for different species/ intermediates.

3.3 Intermediate capture from *S. lasaliensis* ACP12(S970A)

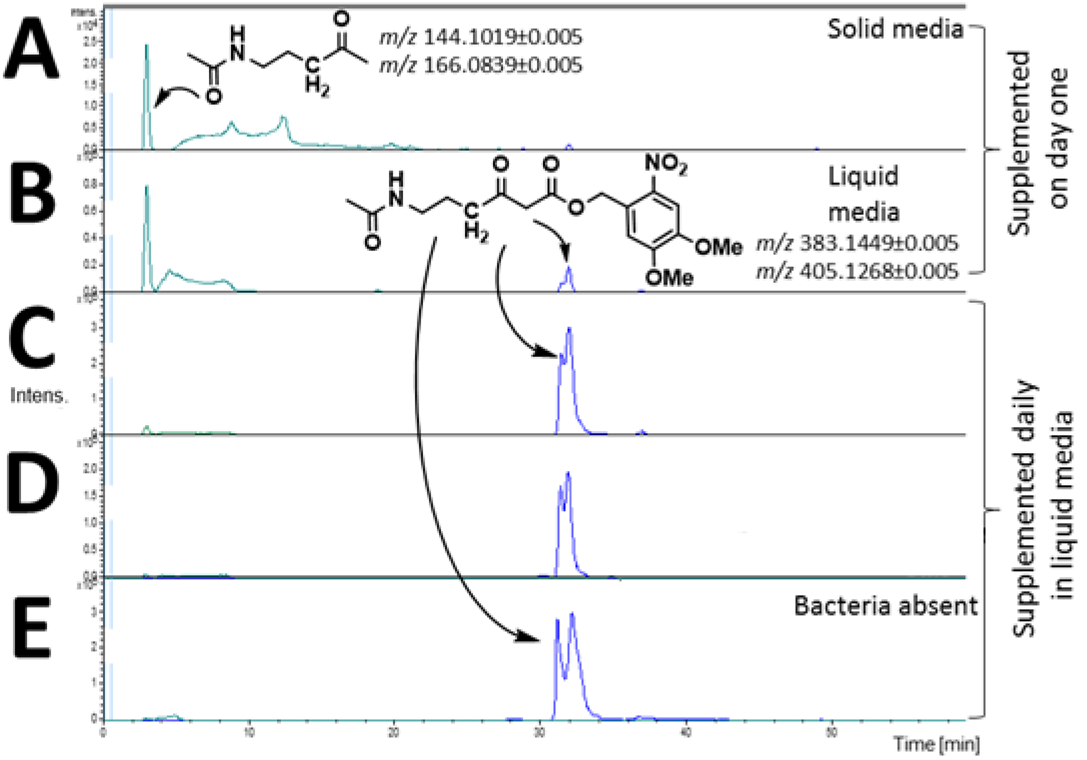

Fig. 3S UPLC-HRMS analyses of organic extracts of *S. lasaliensis* ACP12(S970A) not subjected to photolysis. Extracted ion chromatograms showing: **A)** spontaneous hydrolysis and decarboxylation of photolabile probe **4** over 5 days when the strain was grown on solid MYM containing **4** (2.5 mM); **B)** hydrolysis of **4** over 5 days when the strain was grown in liquid MYM containing **4** in 2.5 mM concentration from day 1 of fermentation; **C)** and **D)**: poor hydrolysis of **4** over 5 days when the strain was grown in liquid MYM and was supplemented daily with small aliquots of **4**; E) poor hydrolysis of **4** over 5 days in liquid MYM (no bacteria present) when supplemented daily in small aliquots.

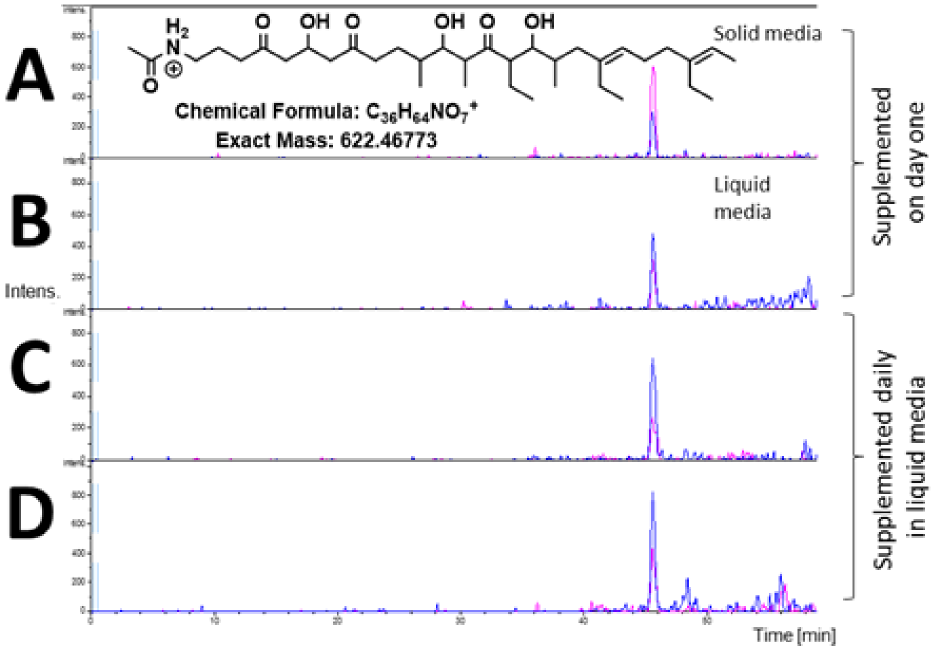

Fig. 4S (above) and 5S (below) UPLC-HRMS analyses of organic extracts of *S. lasaliensis* ACP12(S970A) not subjected to photolysis. Extracted ion chromatograms (shown) revealed the presence of chemically captured undecaketide **6** (above) and dodecaketide **7** (below) intermediates as a result of the hydrolysis of **4** in different conditions. These species have been previously detected and characterised. [3, 4]

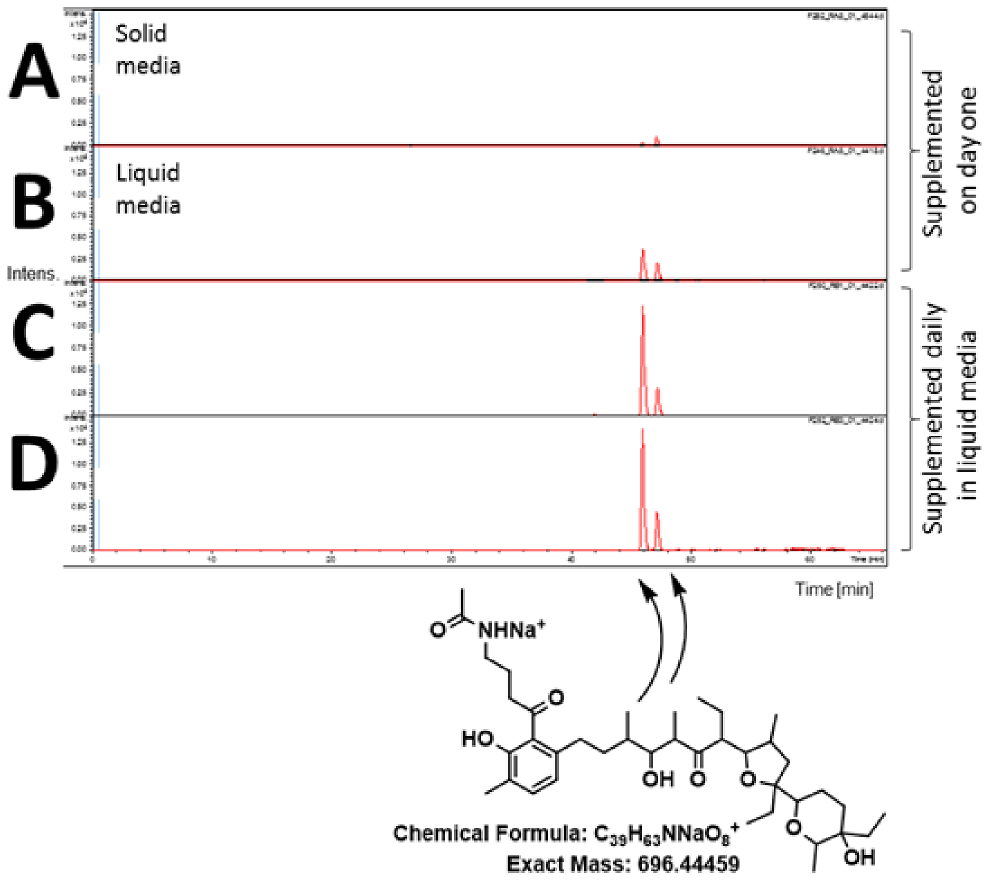

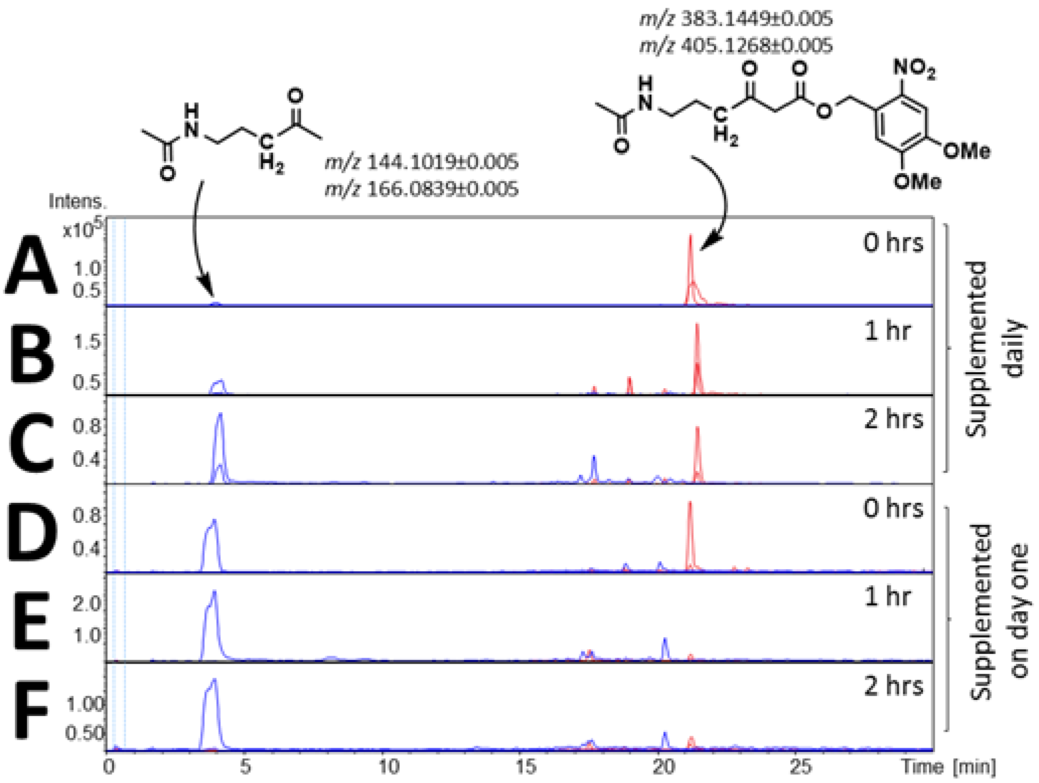

Fig. 6S UPLC-HRMS analyses of organic extracts of *S. lasaliensis* ACP12(S970A) subjected to photolysis of probe **4** revealed irradiation time- dependent formation of active probe **2** (ultimately decarboxylating to *m/z* 144, extracted ion chromatograms shown for all species) [1-3] in MYM liquid cultures: **A** and **D-** no irradiation**;** **B** and **E**- one hour daily irradiation of strain cultured in liquid MYM; **C** and **F**- two hour daily irradiation of strain cultured in liquid MYM.

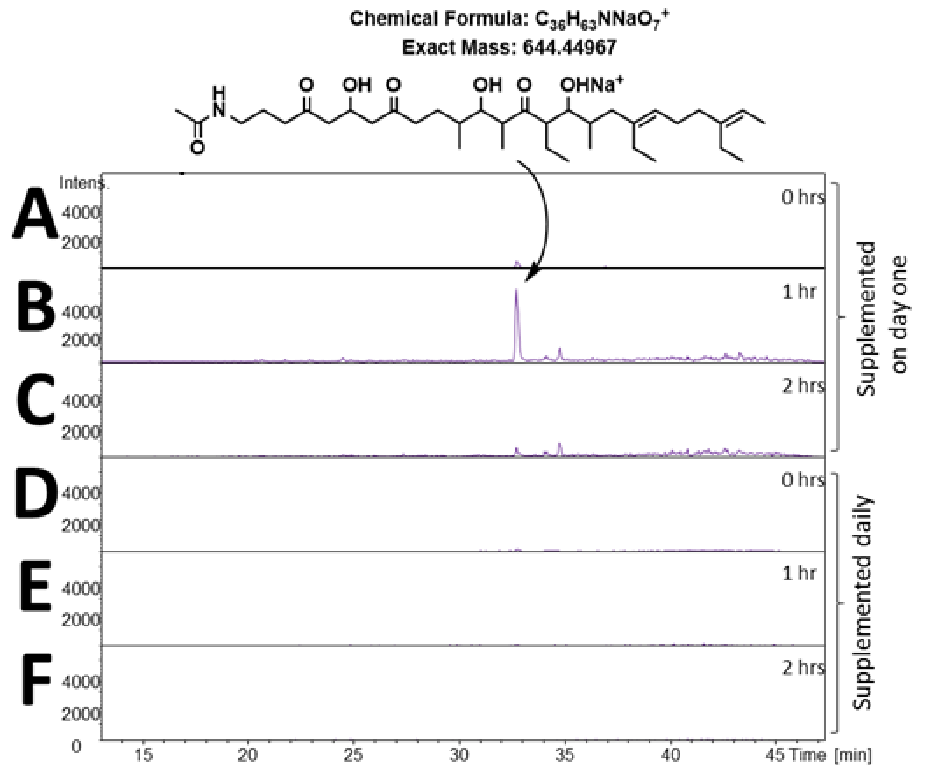

Fig. 7S (above) **and 8S** (below) UPLC-HRMS analyses of organic extracts of *S. lasaliensis* ACP12(S970A) grown in liquid MYM and subjected to photolysis of **4** revealed the presence of chemically captured undecaketide **9** (above) and dodecaketide **7** (below) intermediates (extracted ion chromatograms shown).[3]

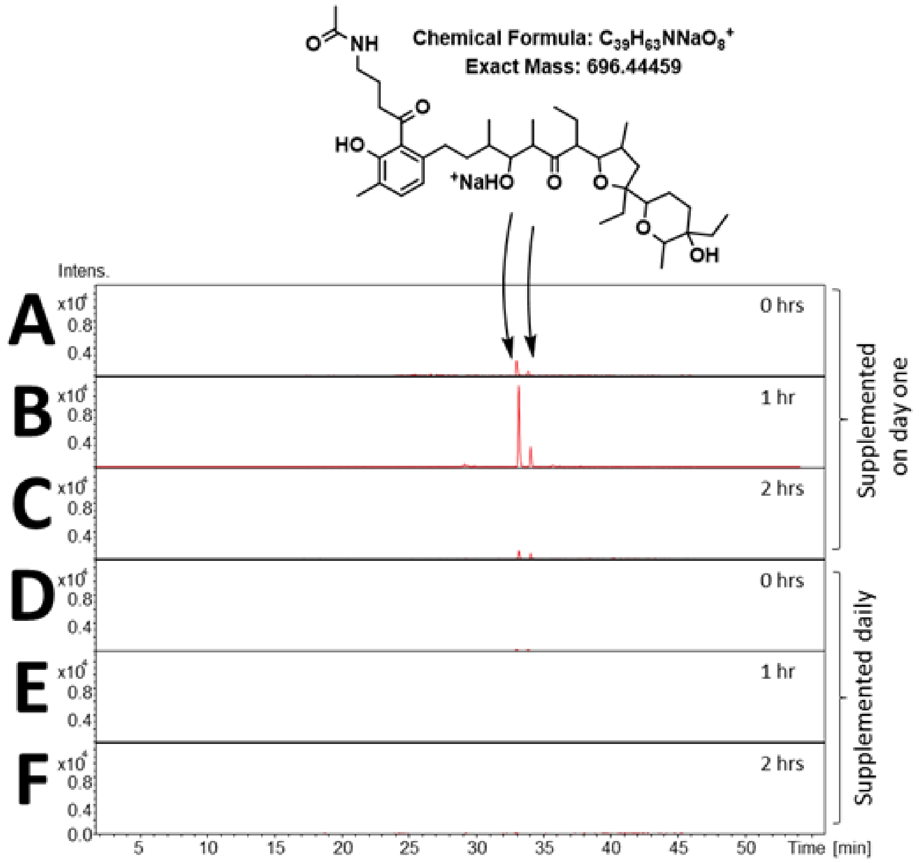

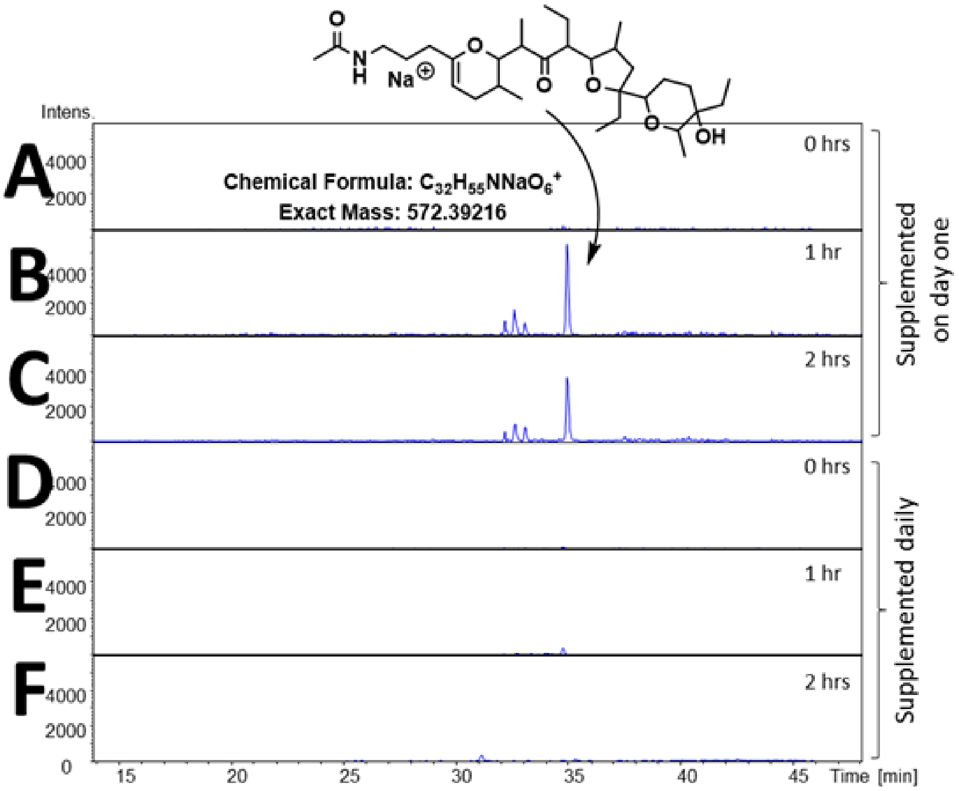

Fig. 9S (above) and **10S** (below). UPLC-HRMS analyses of organic extracts of *S. lasaliensis* ACP12(S970A) grown in liquid MYM and subjected to photolysis of **4** revealed the presence of the putative captured nonaketide **10** (above) and a closely related species **15** (below, extracted ion chromatograms shown for both species). Both **10** and **15** were fragmented and identified as polyether derivatives (see Fig. 2B in main text and Fig. 11S).

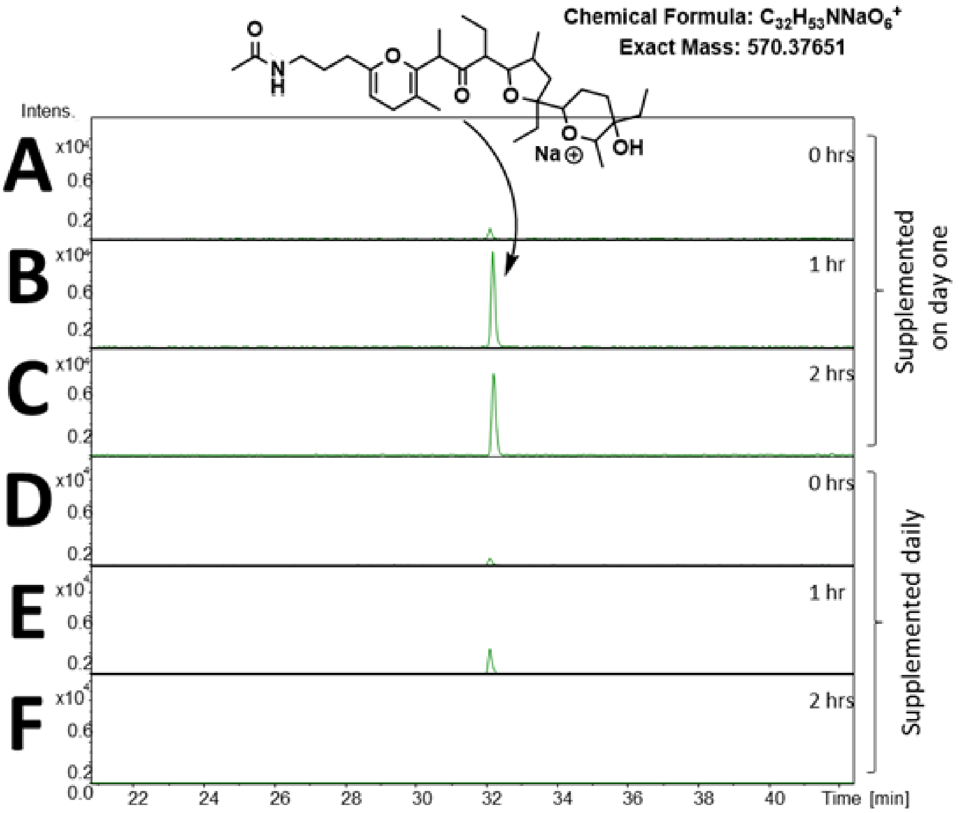

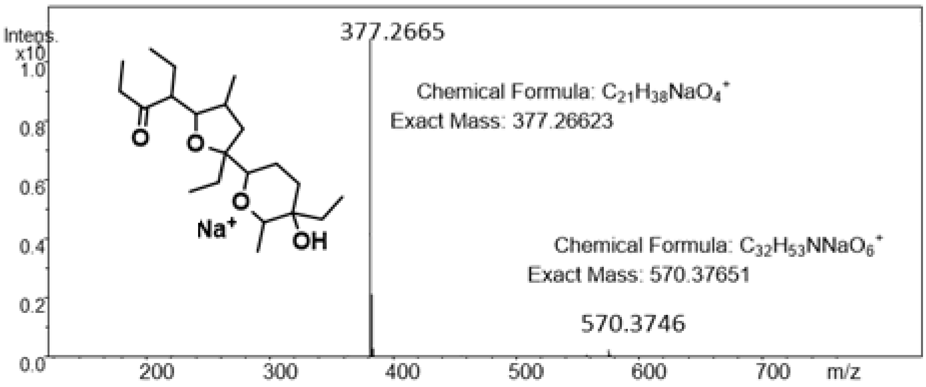

Fig. 11S HRMS^2 analyses of **15** shows the *m/z* 377 fragment typical of lasalocid A derivatives. [3, 4, 5]

4 NMR spectra

4.1 ^{1}H- and ^{13}C-NMR of 4,5-dimethoxy-2-nitrobenzyl 6-acetamido-3-oxohexanoate (4)

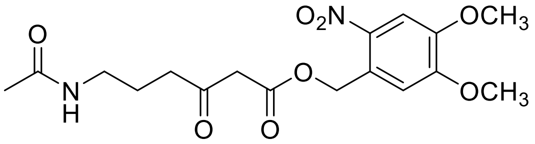

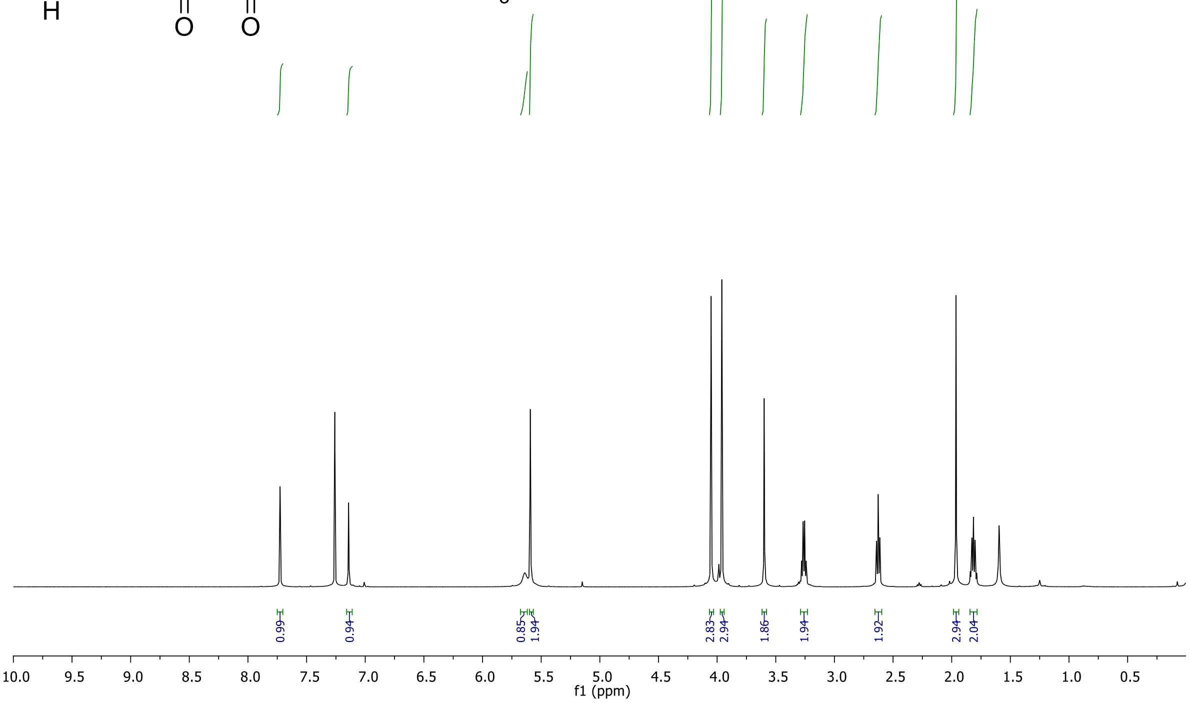

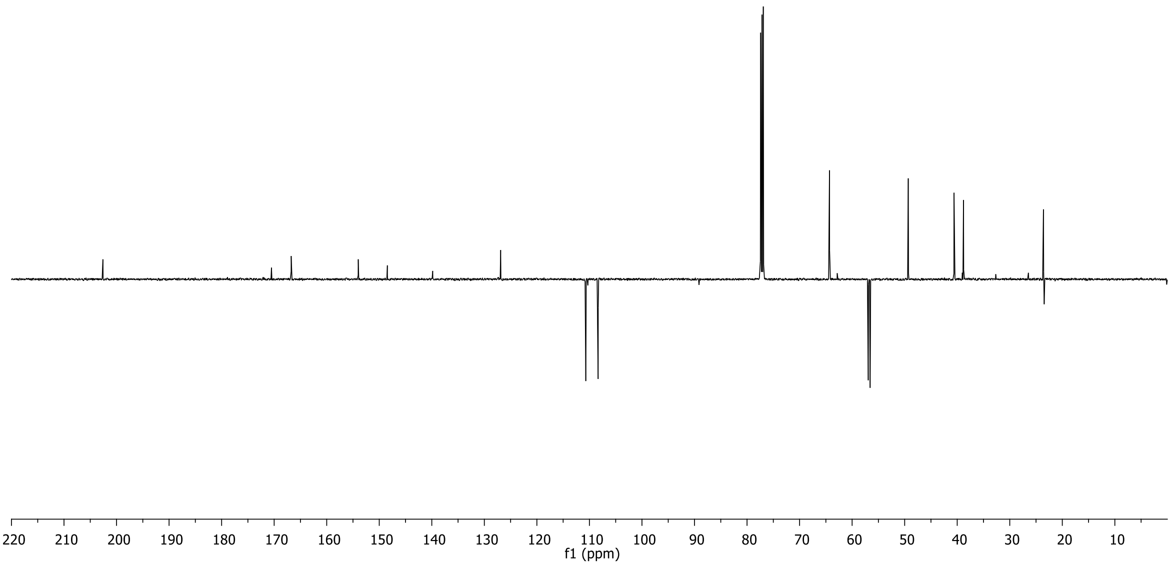

5 References

1. M. Tosin, * L. Betancor, E. Stephens, W. M. Ariel Li, J. B. Spencer, P. F. Leadlay, *ChemBioChem* **2010**, *11*, 539-546.
2. M. Tosin, * Y. Demydchuk, J. S. Parascandolo, C. B. Per, F. J. Leeper, P. F. Leadlay, *Chem. Commun.* **2011**, *47*, 3460-3462.
3. M. Tosin,* L. Smith, P. F. Leadlay, *Angew. Chem. Int. Ed.* **2011**, *50*, 11930-11933.
4. E. Riva, I. Wilkening, S. Gazzola, W. M. Li, L. Smith, P. F. Leadlay, M. Tosin,* *Angew. Chem. Int. Ed. Engl.* **2014**, *53*, 11944-11949.
5. I. Wilkening, S. Gazzola, E. Riva, J. S. Parascandolo, L. Song, L., M. Tosin,* *Chem. Commun.* **2016**, *52*, 10392-10395.
